# Engaging participants in a complex intervention trial in Australian General Practice

**DOI:** 10.1186/1471-2288-8-55

**Published:** 2008-08-13

**Authors:** David Perkins, Mark F Harris, Jocelyn Tan, Bettina Christl, Jane Taggart, Mahnaz Fanaian

**Affiliations:** 1 Centre for Primary Health Care and Equity, School of Public Health and Community Medicine, University of New South Wales, NSW 2052, Australia

## Abstract

**Background:**

The paper examines the key issues experienced in recruiting and retaining practice involvement in a large complex intervention trial in Australian General Practice.

**Methods:**

Reflective notes made by research staff and telephone interviews with staff from general practices which expressed interest, took part or withdrew from a trial of a complex general practice intervention.

**Results:**

Recruitment and retention difficulties were due to factors inherent in the demands and context of general practice, the degree of engagement of primary care organisations (Divisions of General Practice), perceived benefits by practices, the design of the trial and the timing and complexity of data collection.

**Conclusion:**

There needs to be clearer articulation to practices of the benefits of the research to participants and streamlining of the design and processes of data collection and intervention to fit in with their work practices. Ultimately deeper engagement may require additional funding and ongoing participation through practice research networks.

**Trial Registration:**

**Current Controlled Trials **ACTRN12605000788673

## Background

There is an increasing need for complex health service changes to be evaluated in general practice. This is in recognition of the critical role of general practice in the health system and the need for it to respond to new challenges such as the rise of chronic disease and the pressure faced by other sections of the health system such as hospitals. In the 1990s, the majority of funded research in Australian general practice was descriptive [1]. More recently there has been an increased emphasis on conducting randomised trials to produce the high level evidence needed to underpin quality primary health care policy and practice [2]. This has resulted in an increasing number of complex intervention studies commencing in Australian general practice over the past five years.

It is established that such trials need a methodology capable of answering the questions they ask. This includes controlling for other changes which may be occurring and an adequate sample size to adjust for clustering of patients by practices and for loss to follow up [3]. Complex health interventions are built up of several components that may include organisational structures and delivery methods. The UK Medical Research Council framework on evaluating complex interventions emphasises the importance of a stepwise approach to developing and evaluating a complex intervention involving theory, modelling, piloting, and following the trial with an implementation phase [4]. Campbell et al have stressed the importance of the context in which the research is undertaken including health service systems; population characteristics and how these change over time; understanding the problem including the pathways by which problems are caused; the potential for improvement; reviewing barriers to the intervention; optimising components of the intervention and refining the target group to take into account its likelihood of responding to the intervention [5].

There has been little empirical examination of these issues in general practice in Australia. This paper seeks to explore some of the issues related to the recruitment and retention of general practices in such trials using our own experience with a health services trial conducted over the past three years.

## Methods

The trial grew from our previous research on the capacity of general practices to provide quality care for patients with chronic disease that showed a relationship between teamwork within the practice and quality of care [6,7]. Following this, we designed a study to examine the effects of an intervention to increase the team roles of non-GP staff in management of patients with chronic disease. This began with a qualitative study to identify the interventions appropriate for Australian general practice [8]. Focus groups were used to collect data from groups of practice staff: practice nurses, practices managers and receptionists. Semi-structured interviews were conducted by phone with key informants and Chief Executive Officers of Divisions of General Practice (Australian primary care organisations). This research highlighted the importance of key characteristics including leadership, communication protocols, team meetings, information systems and procedures, role definitions and training within the practice.

The intervention consisted of a six month "Teamwork for Chronic Disease Care" program. Based on published evidence, the programme assisted practices to define roles and procedures for practice staff to support GPs in the care of patients with type-2 diabetes, ischaemic heart disease, or hypertension. The program included four elements:

• Practices nominated a non-GP members of staff as chronic disease management (CDM) leader for the program

• The researchers provided education and briefing sessions for GPs and CDM leaders on chronic disease care support, guidelines for structured care for diabetes and cardiovascular disease, and practice systems which asses quality and cost effective teamwork in chronic disease care

• Practice visits by a teamwork facilitator to help practices assess their existing systems, set targets for change, explore barriers and enablers to team working and quality improvement activities using non-GP staff, and addressing processes for improving practice income through providing quality care

• Providing ongoing support through telephone calls and follow-up visits.

**Figure 1:**
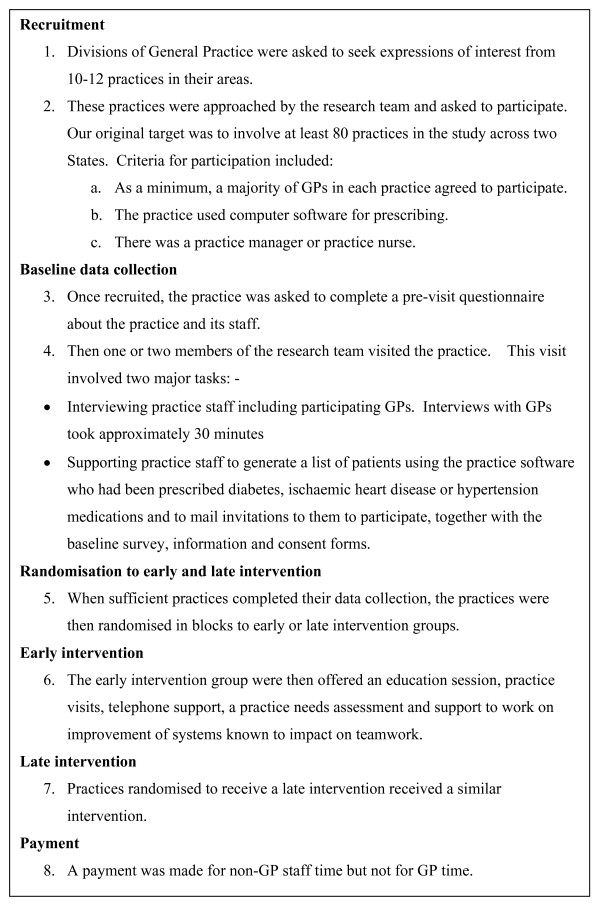
Summary of the "Teamwork" study protocol.

The intervention was piloted in one practice before the project began. However, although the same data collection methods and instruments had been used in our previous study, there was no piloting of the full recruitment, data collection and intervention prior to commencing the study. The University of New South Wales Human Research Ethics Committee approval required arms-length recruitment of both practices and, in particular, patients. Practices could only be approached by Divisions of General Practice to participate and patients could only be approached by their practices. This meant that the researchers could not directly approach either practices or patients. The researchers asked Divisions of General Practice to seek expressions of interest from practices in their territory that passed these to the researchers. Patients who met the inclusion criteria were randomly identified by the practice and approached by mail for their consent. Only when consent was received did the researchers contact patients directly. The study protocol is summarised in figure 1. [see Additional file 1]

### Methodology for this paper

Project evaluation staff conducted telephone interviews with practices that initially expressed interest but did not participate and with those who did participate. We asked about their reasons for participation or non-participation. Practices that agreed to participate but subsequently withdrew after baseline data collection were also asked to give a reason for withdrawing which was recorded. These responses were analysed thematically.

## Results

### The recruitment

Divisions of General Practice were relatively easy to recruit. Initially 20 Divisions expressed interest and 16 participated in the study. For them the main attraction was an interest in the subject matter as this fitted with their core activities of practice support and promoting the use of chronic disease and multidisciplinary care planning. These activities were included in national Division performance indicators. However, Divisions only recruited between one and seven practices each that went on to participate in the study not the larger numbers hoped for.

### Withdrawal after expressing interest

155 practices expressed interest in the study. Of these 87 went on to participate by consenting to take part and providing baseline data. Explanations for not participating after expressing interest were surprisingly diverse. Patient demand and practice workload were the most frequent reasons given and often associated with loss of clinical or administrative staff. This caused practices to refocus on their core business (clinical care) with participation in research being a secondary priority.

Delays between the practice expressing interest and being visited by the research team occurred for a variety of reasons – communication difficulties between Divisions, the research team and practices; logistical difficulties arranging visits; and staff turnover both in practices and the research team. These delays caused a number of practices to lose interest or to become caught up in other developments or activities.

In some cases individual GPs, practice managers or nurses expressed initial interest but could not convince the rest of practice that they should participate. Some larger practices could not secure the agreement of the majority of GPs to take part. Practices faced competing demands from other activities especially preparing practice accreditation or participating in the more generously funded Primary Care Collaboratives Program.

### Withdrawal from the trial after providing baseline data

Of the 87 practices who participated in the initial data collection, 30 subsequently withdrew. Since the research design required completion of baseline data collection before block randomisation by Division, there were long delays for some practices between recruitment, completion of baseline data collection, block randomisation and the start of the intervention. Practices found this frustrating and it led to several withdrawals by practices that had provided baseline data.

Some practices withdrew during the trial when they understood more clearly the extent of data collection required. Since the trial took place over an extended period circumstances changed within particular practices including the dissolution of one practice partnership and the loss of key supporting staff including a practice manager.

### Practices that remained in the trial

57 practices remained in the study and provided data throughout the trial. This provided sufficient power to detect differences in the outcomes between intervention and control groups although it was less than originally hoped for. However the difficulty in recruitment and retention led to a delay in the study by 12 months markedly increasing the costs of the study.

Practices remaining in the trial were more likely than those who did not participate or withdrew to see an opportunity to improve the way in which the practice managed chronic disease, to have a straightforward method of decision making within the practice, and to perceive research as important in improving the quality of care in their practice.

The incentives for practices to participate were largely intrinsic and related to the opportunity to assess the quality of care in their own practice and take steps to improve teamwork. Funding was not a primary consideration and was provided only to defer the costs of practice staff time in data collection and recruitment.

## Discussion

The aim of this paper was to examine the key issues experienced in recruiting and retaining practice involvement in a large complex intervention trial in Australian General Practice and draw out the lessons for the conduct of such studies.

We have learnt some practical lessons for the successful conduct of research in general practice. The requirement of arms-length recruitment of practices through Divisions of General Practice contributed significantly to the difficulties of recruiting general practices and retaining them throughout the trial. This was because we only had an opportunity to explain the study directly to practices after they had been briefed by the Divisions and had expressed interest. In some cases this meant that practices did not have realistic expectations of what was involved in the study and they withdrew either before or after data collection had begun.

We believe that while arms-length recruitment of patients is very appropriate, arms length recruitment of practices interferes with the early establishment of an appropriate relationship between researchers and practitioners. We, as researchers, must be more directly involved in explaining the study to potential participant practices within Divisions especially in explaining the rationale, process, extent of necessary commitments and potential benefits. It is difficult for Divisions to provide this information to practices since they are not primarily research agencies but it is crucial for recruitment and continued engagement. Providing feedback to practices through direct contact, newsletters, and local presentations within their Division were important.

Partnerships with Divisions of General Practice are necessary in the Australian context because of their recognition as gatekeepers to general practice and because direct recruitment of practices is discouraged by most institutional ethics committees. If Divisions are to assist in recruitment (and possibly in the conduct of research) they need to see the research as central to their needs and those of their members. However in our experience, and that of other Australian researchers, engagement of Divisions and successful recruitment is likely to be enhanced if they are formally recognised as research partners and not simply used as a means to obtain practices [9]. We have sought to develop this partnership through better communication of findings of previous research with them and involvement of the Divisions as partners early and throughout the research development process. For intervention research, there may be particular benefits in involving Divisions in the implementation of the intervention (and of course funding them to do so). This is because they are experienced in working with their members, visiting practices, providing training and education and other practice support [10]. It may also allow research studies to more clearly separate the intervention and evaluation arms of their research.

In most research trials, the engagement of practices also needs to be maintained over time. While funding may never be sufficient to act as an incentive on its own, it can reduce the costs of participation. The Australian Association for Academic General Practice has called for similar levels of remuneration to that provided to teaching practices for practices participating in research. Apart from financial measures, more rapid feedback and recognition for their involvement may make it more attractive to GPs. While attaining Continuous Professional Development (CPD) points is an important recognition for some GPs at some points in the CPD cycle, recognition of their continuing involvement in research may also be encouraged by other measures such as invitations to presentations of the findings, formal appointments as research collaborators or as research network members [11,12].

We found it vitally important to minimise the time intervals between expression of interest and practice visits and between recruitment and initiating the intervention within each practice. This required a change in our research protocol to allow earlier contact with the practices and to minimise the delays due to block randomisation. We have also recognised the burden of research participation for general practice and have learnt to minimise the amount of time required of all practice staff, including GPs.

We anticipate that practice recruitment to research will become more, not less, difficult in the medium term due to increasing work pressures, shortage of GPs and other primary care staff and competition for practice involvement from other initiatives and programs.

Those designing and evaluating complex interventions in Australian general practice face very real constraints in recruiting and retaining sufficient practices. This is particularly so for health service intervention studies, such as our study of teamwork, that some GPs may find less attractive than specific therapeutic interventions. Research methodology must respond flexibly to the needs of and demands on practices. Random allocation of practices has methodological advantages to randomisation of individual patients or non-random allocation. However this may act as a significant barrier to engagement of practices even where delayed intervention is offered to the control group of practices. Other designs (such as quasi-experimental designs) are likely to be more acceptable and may need to be considered where they can answer the research questions.

## Conclusion

In this paper we have described some pragmatic and more serious obstacles to conducting intervention research studies in Australian general practices. Overcoming these obstacles is important for the following reasons. In the face of an increased burden of chronic diseases, we need to know if care is most effectively and efficiently organised. In a period of international workforce shortage, both medical, allied health and nursing we need to know how teams with an appropriate skill-mix are able to work together to provide the best possible care. The first challenge in trying to answer these questions is to engage general practice in the research process. Meeting this challenge requires all our creativity and ingenuity.

## Abbreviations

GP: general practitioner; CDM: Chronic disease management; Division: Division of General Practice; CPD: Continuous Professional Development.

## Competing interests

The authors declare that they have no competing interests.

## Authors' contributions

DP drafted the paper. JoT, BC, JaT, and MF undertook the underpinning research and MFH substantially revised the paper. All authors read and approved the final manuscript.

## Pre-publication history

The pre-publication history for this paper can be accessed here:



## Supplementary Material

Additional file 1 Why this paper is important. Click here for file
