# Supplementary material for: Engaging participants in a complex intervention trial in Australian General Practice
Source: BMC Med Res Methodol. 2008 Aug 13;8:55. doi: 10.1186/1471-2288-8-55 (PMC2533668; doi:10.1186/1471-2288-8-55)
Supplement: Additional file 1 — Why this paper is important. [file 1471-2288-8-55-S1.doc]

**Why is this paper important?**

General practices are at the front line of health care and critical in the response to the burden of chronic disease and pressure on the hospital system. However, there is little evidence of the effectiveness of interventions and programmes in general practice.

Attempts to conduct complex intervention trials in settings such as Australian general practice face particular problems. This paper describes both the problems encountered and the solutions found in recruiting and retaining general practices in a complex intervention trial.

The trial is unusual since it addresses the issue of teamwork and the contribution of non-GP staff in the provision of quality care for patients with chronic diseases. This paper is of interest to researchers who struggle with the difficulties of conducting such trials but will also be of interest to policy makers who seek evidence about the impact of their programmes and may need to support general practices who participate in such research programmes.

The paper provides lessons that can inform researchers planning to conduct such complex intervention studies and raises issues for policy makers in relation to how such research can be supported in the future.
